# Extensive Mucocutaneous Verruca Vulgaris in a Nonimmunocompromised Patient

**DOI:** 10.5005/jp-journals-10005-1084

**Published:** 2011-04-15

**Authors:** Vela D Desai, Rajeev Sharma, Durgesh N Bailoor

**Affiliations:** 1 Professor and Head, Department of Oral Medicine and Radiology, Jaipur Dental College, Jaipur, Rajasthan, India; 2 Senior Lecturer, Department Oral Medicine and Radiology, Jaipur Dental College, Jaipur, Rajasthan, India; 3 Professor, Department of Oral Medicine and Radiology, Karnavati Dental College, Ahmedabad, Gujarat, India

**Keywords:** Verruca vulgaris, Warts, Human papilloma virus.

## Abstract

Papilloma virus infections of the oral cavity have been long recognized with various clinical expressions characterized as verruca vulgaris, Heck’s disease, multiple papilloma and condyloma acuminata. In this paper, we are highlighting a case of verruca vulgaris involving the oral cavity with extensive skin lesions in a nonimmunocompromised 9-year-old boy. Different treatment modalities are discussed in this article.

## INTRODUCTION

Verruca vulgaris is a relatively uncommon oral lesion which is caused by an infectious agent HPV (human papilloma virus). Verruca vulgaris (VV) of oral mucosa is rare and is typically a childhood problem. The lesions of verruca vulgaris are circumscribed, firm, elevated papule with papillomatous hyperkeratotic surface. They occur most commonly on the dorsal aspect of the fingers and hand. They are also found on sole of feet. Lesions of verucca occur extensively in immunocompromised host. Here, we present a case of severe form of verruca in a healthy individual.

## CASE REPORT

**Fig. 1:**
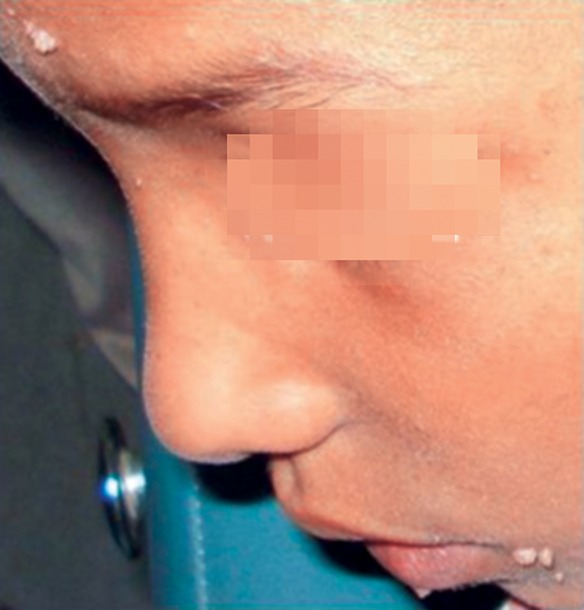
A 9-year-old boy presenting with papillary growths on the forehead, inner canthus of eye, left angle of mouth and on vermilion border of lip

**Figs 2A and B:**
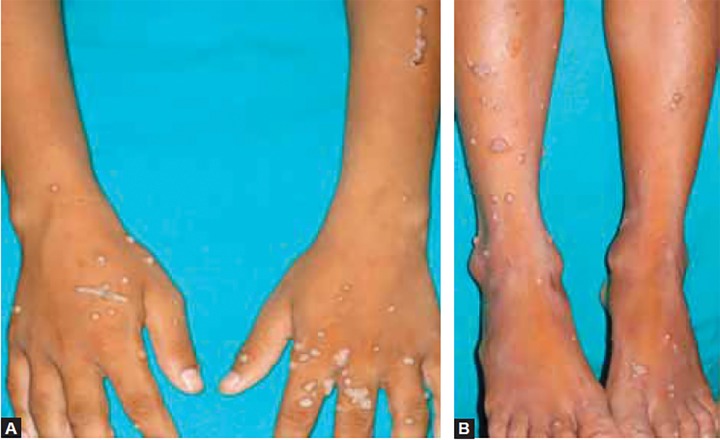
Multiple grayish white lesions on the hands and legs

**Fig. 3:**
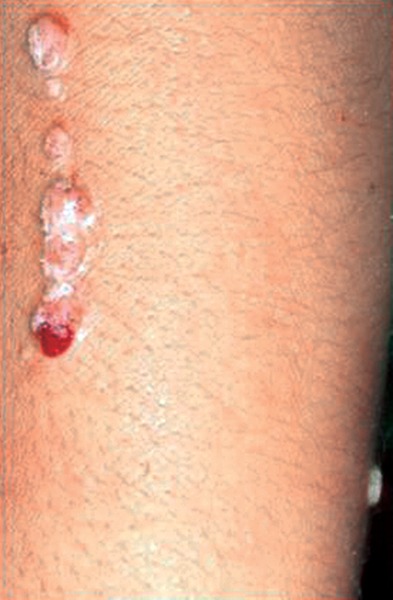
Coalesced linear occurring lesions showing bleeding

A 9-year-old boy reported to the Department of Oral Medicine, Radiology and Diagnosis with multiple, small, whitish growth on his legs, hands, fingers and face (Fig. 1). The first symptom of this appeared 2 year ago. Lesions were asymptomatic except for slight occasional itching. Similar type of lesion also had been noticed in his elder brother and peers, which regressed spontaneously without any treatment. Systemic review was noncontributory. Patient neither suffer from any long-term illness in the past nor was on any medications. On extraoral examination, numerous keratotic, well-defined whitish colored, papules varying in size from 2 to 4 mm were found on dorsum of hand, feet, face, chin and angle of mandible (Figs 1 to 2B). On left hand they were found in groups of more than 20 in number. Some of the lesions on left hand were in a linear fashion and exhibited Koebner phenomenon. Mild discharge of blood was noticed on scraping the lesion (Fig. 3).

**Fig. 4:**
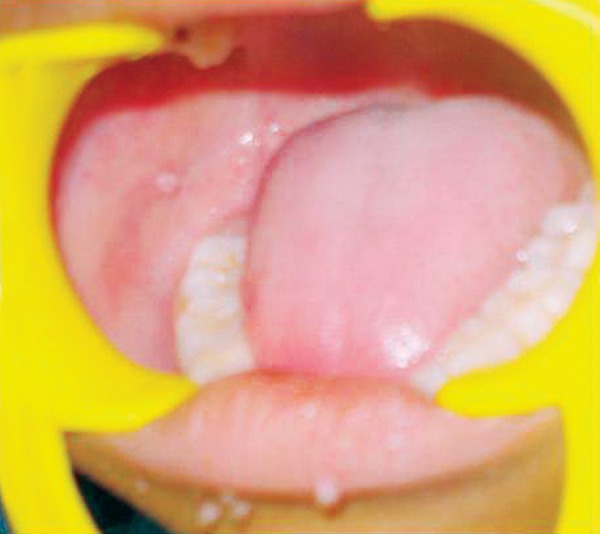
Intraorally small flat lesion on right buccal mucosa

Examination of the oral cavity revealed whitish solitary lesion on right buccal mucosa with finger like projection and multiple small (4-5 in number) lesions were also appreciable on lower labial mucosa and on alveolar ridge in the region of lower left lateral incisor and canine (Fig. 4). On palpation they were firm in consistency and nontender. Patient had an otherwise good oral hygiene. Examination of other systems was noncontributory. A provisional diagnosis of verruca vulgaris was made.

**Figs 5A and B:**
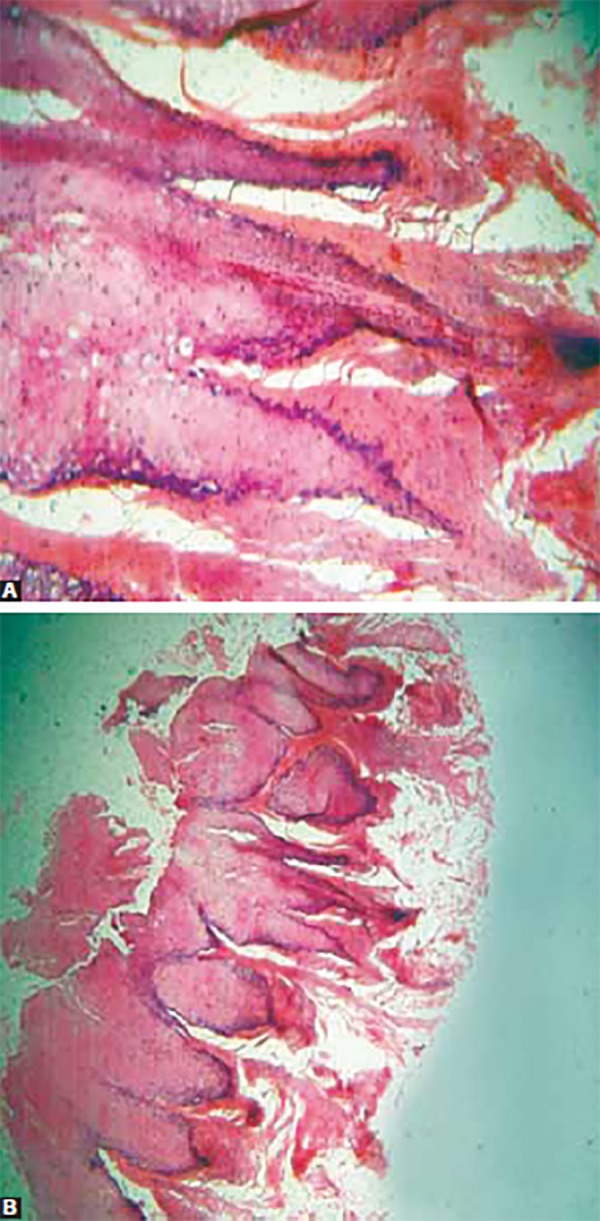
Histopathology showing the classical lesion of verucca vulgaris

The patient underwent a complete blood investigation including Elisa test, which were within normal range. Excisional biopsy was performed with the patients parents consent (as he was a minor) from two different sites, i.e. dorsum of hand and intraorally from the right buccal mucosa. Histopathological examination revealed acanthosis with proliferative, hyperkeratotic, stratified squamous epithelium showing finger like projection and elongated rate pegs converging towards the center, producing cupping effect suggestive of verruca vulgaris. There were foci of vacuoled cells referred to as koilocytotic cells. Vertical tier of parakeratotic cells and foci of clumped keratohyalin granules were also seen (Figs 5A and B). Patient underwent a thorough oral prophylaxis only.

## DISCUSSION

A variety of papillary lesions occur in the oral cavity and while many can be differentiated on the basis of histopathological features, they may closely resemble one another clinically^1^. Verruca vulgaris, condyloma acuminate, Heck’s disease, squamous papilloma are all local papillary lesions that share similar clinical feature yet their microscopic patterns differ.^1,2^ Verruca vulgaris commonly occurs in children and usually regresses spontaneously over a period of time.^2,3^ Papilloma virus type 2 and 4 are the most prevalent isolates from cutaneous warts, and these same viral genotypes are identifiable when verruca valgaris arise on the lip or on mucous membrane. Whereas condylomas are usually diagnosed in teenagers and young adults and are more frequently encountered in the mucosa membranes of the anogenital region, it may also be seen in the oral cavity perhaps arising therein as a consequence of oragenital sexual transmission.^1^ They are bigger in size and appear pink to red as a results of less keratinization.

The case herein represent classical form of oral verruca vulgaris with extensive multiple skin involvement. Such type of exacerbated involvement is usually observed in immunocompromised host.^4^ There have been very few reported cases of verruca vulgaris in a nonimmuno-compromised patients. Thus, presented case is unique as there was no underlying immune supression.

From clinical observation, we found that the lesions were mainly located on the buccal mucosa, lower labial mucosa, also autoinoculation from a lesion located on hand has been observed. A slightly higher incidence was encountered for OVV in children and young adults and differs from other reports.^4,5^ Kirchner has speculated that a certain kind of lesion is more frequently found before puberty and links its presence to a genetic defect that retards the establishment of an effective defense mechanism until puberty leaving some children more susceptible than others to the infection.^6^ Verruca vulgaris may not require any treatment, but is done for cosmetic purposes and to prevent their spread.^7^

Most people develop an immune response that causes verruca vulgaris to vanish by themselves.^8^ The patient’s sibling’s lesions vanished in a similar way and in the present case also, no treatment was initiated as the patient denied any treatment.

Skin verruca vulgaris is treated effectively by liquid nitrogen, cryotheraphy, conservative surgical excision or curettage or topical application of keratinolytic agents containing salicylic acid and lactic acid.^7,8^ Oral lesions are successfully surgically excised or they may be destroyed by laser, cryotheraphy or electrosurgery. Recurrence is seen in a small proportion of treated case. Verruca vulgaris does not transform into malignancy with or without treatment and two third will disappear spontaneously within 2 years especially in children.^7^ Detailed genetic study should be carried out to rule out any genetic origin in all the suspected cases.

## CONCLUSION

Due to paucity of cases of oral verruca vulgaris being reported, information regarding malignant potential is limited. In the current case despite an extensive skin lesion there was no evidence of underlying disease (AIDS/ dysplasia on histopathology). On basis of a single case, it is very difficult to infer that they may occur in nonimmuno-compromised patient and have no malignant potential. More studies need to be carried out to corroborate that they occur in immunocompromised patient. Any patient with presence of extensive verruca vulgaris lesion must be eyed with suspicion of AIDS/compromised immune system or internal malignancy and thorough blood investigation including ELISA/immunohistochemistry/*in situ* hybridization/ southern blot hybridization should be carried out. Fortunately in our case patient did not have any of this life threatening condition. The association of oral lesion with HPV should be extensively examined to clarify the pathogenesis of HPV infection in oral cavity.
